# Ethyl 4-hy­droxy-2,6-diphenyl-1-[2-(piperidin-1-yl)acet­yl]-1,2,5,6-tetra­hydro­pyridine-3-carboxyl­ate

**DOI:** 10.1107/S1600536810026619

**Published:** 2010-07-14

**Authors:** G. Aridoss, S. Sundaramoorthy, D. Velmurugan, K. S. Park, Y. T. Jeong

**Affiliations:** a Department of Image Science and Engineering, Pukyong National University, Busan 608-739, Republic of Korea; b Centre of Advanced Study in Crystallography and Biophysics, University of Madras, Guindy Campus, Chennai 600 025, India

## Abstract

In the title compound, C_27_H_32_N_2_O_4_, the piperidine and tetra­hydro­pyridine rings adopt chair and half-chair conformations, respectively. The dihedral angle between the two phenyl rings is 32.9 (1)°. The mol­ecular structure is stabilized by a strong intra­molecular O—H⋯O hydrogen bond, generating an *S*(6) motif. In the crystal, inter­molecular C—H⋯O inter­actions form a ribbon-like structure along the *a* axis.

## Related literature

For the biological activity of piperidines, see: Aridoss *et al.* (2008 ▶, 2010 ▶). For related structures, see: Subha Nandhini *et al.* (2003 ▶); Aridoss *et al.* (2009*a* ▶,*b* ▶); Parkin *et al.* (2004 ▶). For ring conformational analysis, see: Cremer & Pople (1975 ▶); Nardelli (1983 ▶).
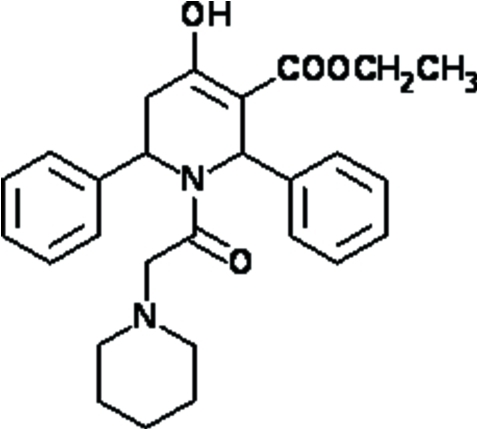


## Experimental

### 

#### Crystal data

C_27_H_32_N_2_O_4_
*M* *_r_* = 448.55
Monoclinic, 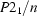

*a* = 10.7936 (6) Å
*b* = 9.6752 (6) Å
*c* = 23.2335 (13) Å
β = 93.213 (3)°
*V* = 2422.5 (2) Å^3^
*Z* = 4
Mo *K*α radiation
μ = 0.08 mm^−1^
*T* = 292 K
0.25 × 0.23 × 0.20 mm

#### Data collection

Bruker SMART APEXII area-detector diffractometer
Absorption correction: multi-scan (*SADABS*; Bruker, 2008 ▶) *T*_min_ = 0.979, *T*_max_ = 0.984
21907 measured reflections
5870 independent reflections
3631 reflections with *I* > 2σ(*I*)
*R*_int_ = 0.025

#### Refinement

*R*[*F* ^2^ > 2σ(*F* ^2^)] = 0.058
*wR*(*F* ^2^) = 0.193
*S* = 1.05
5870 reflections
299 parameters
1 restraint
H-atom parameters constrained
Δρ_max_ = 0.58 e Å^−3^
Δρ_min_ = −0.37 e Å^−3^

### 

Data collection: *APEX2* (Bruker, 2008 ▶); cell refinement: *SAINT* (Bruker, 2008 ▶); data reduction: *SAINT*; program(s) used to solve structure: *SHELXS97* (Sheldrick, 2008 ▶); program(s) used to refine structure: *SHELXL97* (Sheldrick, 2008 ▶); molecular graphics: *ORTEP-3* (Farrugia, 1997 ▶); software used to prepare material for publication: *SHELXL97* and *PLATON* (Spek, 2009 ▶).

## Supplementary Material

Crystal structure: contains datablocks global, I. DOI: 10.1107/S1600536810026619/ci5118sup1.cif

Structure factors: contains datablocks I. DOI: 10.1107/S1600536810026619/ci5118Isup2.hkl

Additional supplementary materials:  crystallographic information; 3D view; checkCIF report

## Figures and Tables

**Table 1:** Hydrogen-bond geometry (Å, °)

*D*—H⋯*A*	*D*—H	H⋯*A*	*D*⋯*A*	*D*—H⋯*A*
O2—H2⋯O3	0.82	1.88	2.598 (3)	145
C2—H2*B*⋯O1^i^	0.97	2.43	3.286 (3)	148
C10—H10⋯O1^ii^	0.93	2.50	3.427 (3)	177

Symmetry codes: (i) 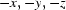; (ii) 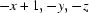.

**Fig. 1.**
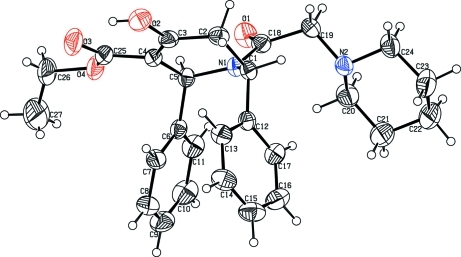
The molecular structure of the title compound, showing displacement ellipsoids drawn at the 30% probability level.

**Fig. 2.**
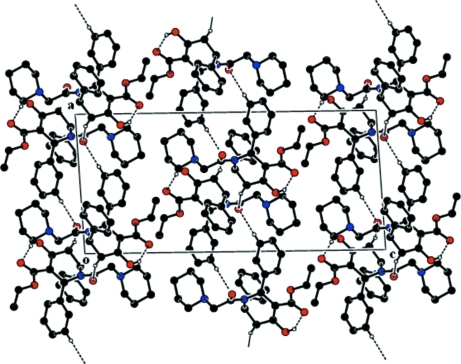
The crystal packing of the title compound viewed down the b axis. For clarity, H atoms not involved in hydrogen bonding (dashed lines) have been omitted.

## supplementary crystallographic information

### Comment

Piperidine class of compounds is the valued heterocyclic compounds in the field of medicinal chemistry. We are interested in the title compound as similar type of derivatives have been found to exhibit remarkable antibacterial and antitumor properties (Aridoss *et al.*, 2008, 2010). Recently, we have reported the crystal structures of few tetrahydropyridine derivatives (Aridoss *et al.*, 2009*a*, 2009*b*). As part of our ongoing studies on establishing the conformation of the compounds through X-ray studies, we herein report the crystal structure of the title compound.

In the present structure, the piperidine ring adopts a chair conformation and the tetrahydropyridine ring is in a half-chair conformation. The sum of the bond angles around atoms N1 (357.8 (9)°) and N2 (329.6 (6)°) indicate *sp*^2^ and *sp*^3^ hybridizations, respectively. The puckering parameters (Cremer & Pople, 1975) and the smallest displacement asymmetry parameters (Nardelli, 1983) for the piperidine/tetrahydropyridine ring are q_2_ = 0.022 (3)/0.354 (4) Å, q_3_ = -0.572 (3)/0.293 (2) Å, Q_T_ = 0.572 (3)/0.459 (2) Å, and θ = 179.2 (3)/50.4 (2)°. The dihedral angle between the two phenyl rings is 32.9 (1)°. The piperidine and tetrahydropyridine rings are connected by the ethanone. The ethyl acetate group shows an extended conformation [C27—C26—O4—C25 = -116.4 (5)°]. The molecular structure is stabilized by a strong O—H···O hydrogen bond, wherein, atom O2 acts as a donor to O3, generating an *S*(6) motif.

Atoms C2 and C10 act as donors to form hydrogen bonds with atom O1 as an aceptor. In the crystal structure, the molecules at (*x*,*y*,*z*),  (-*x*,-*y*,-*z*) and (1 - *x*,-*y*,-*z*) are linked into a ribbon-like structure along the *a* axis by C—H···O hydrogen bonds; the ribbons contain *R_2_^2^*(12) and *R_2_^2^*(16) ring motifs.

### Experimental

A mixture of piperidine (1 equiv.), *N*-chloroacetyl-3-carboxyethyl-2,6-diphenylpiperidin-4-one (1 equiv.) and anhydrous potassium carbonate (2 equiv.) in benzene was refluxed on an oil bath until its completion (Aridoss *et al.*, 2010). The crude product obtained after usual work-up upon purification by column chromatography followed by re-crystallization in ethanol yielded fine crystals.

### Refinement

H atoms were positioned geometrically (O–H = 0.82 Å and C–H = 0.93–0.98 Å) and allowed to ride on their parent atoms, with 1.5*U*_eq_(C) for methyl H and 1.2*U*_eq_(C) for other H atoms.

### Crystal data

**Table:**

C_27_H_32_N_2_O_4_	*F*(000) = 960
*M**_r_* = 448.55	*D*_x_ = 1.230 Mg m^−^^3^
Monoclinic, *P*2_1_/*n*	Mo *K*α radiation, λ = 0.71073 Å
Hall symbol: -P 2yn	Cell parameters from 1525 reflections
*a* = 10.7936 (6) Å	θ = 1.8–28.3°
*b* = 9.6752 (6) Å	µ = 0.08 mm^−^^1^
*c* = 23.2335 (13) Å	*T* = 292 K
β = 93.213 (3)°	Block, colourless
*V* = 2422.5 (2) Å^3^	0.25 × 0.23 × 0.20 mm
*Z* = 4	

### Data collection

**Table:**

Bruker SMART APEXII area-detector diffractometer	5870 independent reflections
Radiation source: fine-focus sealed tube	3631 reflections with *I* > 2σ(*I*)
graphite	*R*_int_ = 0.025
ω and φ scans	θ_max_ = 28.3°, θ_min_ = 1.8°
Absorption correction: multi-scan (*SADABS*; Bruker, 2008)	*h* = −14→14
*T*_min_ = 0.979, *T*_max_ = 0.984	*k* = −12→12
21907 measured reflections	*l* = −30→29

### Refinement

**Table:**

Refinement on *F*^2^	Primary atom site location: structure-invariant direct methods
Least-squares matrix: full	Secondary atom site location: difference Fourier map
*R*[*F*^2^ > 2σ(*F*^2^)] = 0.058	Hydrogen site location: inferred from neighbouring sites
*wR*(*F*^2^) = 0.193	H-atom parameters constrained
*S* = 1.05	*w* = 1/[σ^2^(*F*_o_^2^) + (0.0946*P*)^2^ + 0.5359*P*] where *P* = (*F*_o_^2^ + 2*F*_c_^2^)/3
5870 reflections	(Δ/σ)_max_ = 0.001
299 parameters	Δρ_max_ = 0.58 e Å^−^^3^
1 restraint	Δρ_min_ = −0.37 e Å^−^^3^

### Special details

**Table:**

Geometry. All e.s.d.'s (except the e.s.d. in the dihedral angle between two l.s. planes) are estimated using the full covariance matrix. The cell e.s.d.'s are taken into account individually in the estimation of e.s.d.'s in distances, angles and torsion angles; correlations between e.s.d.'s in cell parameters are only used when they are defined by crystal symmetry. An approximate (isotropic) treatment of cell e.s.d.'s is used for estimating e.s.d.'s involving l.s. planes.
Refinement. Refinement of *F*^2^ against ALL reflections. The weighted *R*-factor *wR* and goodness of fit *S* are based on *F*^2^, conventional *R*-factors *R* are based on *F*, with *F* set to zero for negative *F*^2^. The threshold expression of *F*^2^ > σ(*F*^2^) is used only for calculating *R*-factors(gt) *etc*. and is not relevant to the choice of reflections for refinement. *R*-factors based on *F*^2^ are statistically about twice as large as those based on *F*, and *R*- factors based on ALL data will be even larger.

### Fractional atomic coordinates and isotropic or equivalent isotropic displacement parameters (Å^2^)

**Table:**

	*x*	*y*	*z*	*U*_iso_*/*U*_eq_	
C27	0.3740 (4)	−0.0409 (8)	0.2481 (2)	0.206 (3)	
H27A	0.4323	−0.0751	0.2218	0.308*	
H27B	0.3930	−0.0787	0.2857	0.308*	
H27C	0.3791	0.0581	0.2498	0.308*	
C1	0.08942 (18)	0.31589 (19)	0.01812 (8)	0.0470 (4)	
H1	0.0592	0.3326	−0.0218	0.056*	
C2	−0.02445 (18)	0.2987 (2)	0.05340 (9)	0.0539 (5)	
H2A	−0.0605	0.3888	0.0600	0.065*	
H2B	−0.0859	0.2436	0.0317	0.065*	
C3	0.00608 (18)	0.2312 (2)	0.10962 (9)	0.0528 (5)	
C4	0.10915 (17)	0.1540 (2)	0.12035 (8)	0.0492 (5)	
C5	0.20372 (17)	0.1306 (2)	0.07547 (8)	0.0455 (4)	
H5	0.2104	0.0302	0.0711	0.055*	
C6	0.33462 (17)	0.1815 (2)	0.09306 (8)	0.0467 (4)	
C7	0.35769 (19)	0.2807 (3)	0.13484 (9)	0.0573 (5)	
H7	0.2915	0.3222	0.1521	0.069*	
C8	0.4785 (2)	0.3197 (3)	0.15163 (11)	0.0733 (7)	
H8	0.4926	0.3864	0.1801	0.088*	
C9	0.5763 (2)	0.2598 (3)	0.12621 (13)	0.0821 (8)	
H9	0.6571	0.2854	0.1374	0.098*	
C10	0.5550 (2)	0.1619 (3)	0.08418 (14)	0.0822 (8)	
H10	0.6215	0.1217	0.0667	0.099*	
C11	0.4350 (2)	0.1225 (3)	0.06762 (11)	0.0645 (6)	
H11	0.4215	0.0557	0.0392	0.077*	
C12	0.17351 (17)	0.4373 (2)	0.03603 (8)	0.0462 (4)	
C13	0.1481 (2)	0.5271 (2)	0.08002 (10)	0.0580 (5)	
H13	0.0799	0.5107	0.1018	0.070*	
C14	0.2225 (2)	0.6412 (3)	0.09232 (12)	0.0725 (7)	
H14	0.2044	0.7001	0.1223	0.087*	
C15	0.3227 (3)	0.6673 (3)	0.06033 (13)	0.0771 (7)	
H15	0.3720	0.7446	0.0682	0.093*	
C16	0.3502 (2)	0.5787 (3)	0.01649 (13)	0.0734 (7)	
H16	0.4186	0.5956	−0.0051	0.088*	
C17	0.2763 (2)	0.4647 (2)	0.00461 (10)	0.0591 (5)	
H17	0.2957	0.4052	−0.0250	0.071*	
C18	0.15252 (19)	0.0927 (2)	−0.02602 (9)	0.0522 (5)	
C19	0.0955 (2)	0.1375 (2)	−0.08475 (9)	0.0581 (5)	
H19A	0.0885	0.0571	−0.1097	0.070*	
H19B	0.0122	0.1714	−0.0798	0.070*	
C20	0.2914 (2)	0.1989 (3)	−0.12236 (11)	0.0752 (7)	
H20A	0.2891	0.1152	−0.1454	0.090*	
H20B	0.3334	0.1777	−0.0854	0.090*	
C21	0.3637 (3)	0.3090 (4)	−0.15246 (13)	0.0973 (10)	
H21A	0.3713	0.3905	−0.1282	0.117*	
H21B	0.4466	0.2752	−0.1585	0.117*	
C22	0.2998 (3)	0.3470 (4)	−0.20973 (12)	0.0988 (10)	
H22A	0.3428	0.4237	−0.2267	0.119*	
H22B	0.3018	0.2690	−0.2359	0.119*	
C23	0.1672 (3)	0.3868 (4)	−0.20086 (12)	0.0906 (9)	
H23A	0.1240	0.4028	−0.2380	0.109*	
H23B	0.1658	0.4722	−0.1790	0.109*	
C24	0.1010 (2)	0.2751 (3)	−0.16913 (10)	0.0691 (6)	
H24A	0.0169	0.3049	−0.1631	0.083*	
H24B	0.0964	0.1920	−0.1925	0.083*	
C25	0.1255 (2)	0.0802 (3)	0.17438 (9)	0.0638 (6)	
C26	0.2522 (3)	−0.0805 (4)	0.22867 (13)	0.1089 (12)	
H26A	0.1940	−0.0565	0.2574	0.131*	
H26B	0.2487	−0.1797	0.2226	0.131*	
N1	0.15713 (15)	0.18363 (16)	0.01878 (6)	0.0461 (4)	
N2	0.16459 (16)	0.24368 (19)	−0.11332 (7)	0.0543 (4)	
O1	0.19378 (17)	−0.02442 (17)	−0.02074 (7)	0.0726 (5)	
O2	−0.08101 (14)	0.2495 (2)	0.14807 (7)	0.0731 (5)	
H2	−0.0613	0.2069	0.1777	0.110*	
O3	0.05991 (18)	0.0935 (3)	0.21512 (7)	0.0941 (7)	
O4	0.21980 (15)	−0.0091 (2)	0.17514 (7)	0.0785 (5)	

### Atomic displacement parameters (Å^2^)

**Table:**

	*U*^11^	*U*^22^	*U*^33^	*U*^12^	*U*^13^	*U*^23^
C27	0.141 (4)	0.309 (8)	0.158 (4)	−0.045 (4)	−0.073 (3)	0.136 (5)
C1	0.0501 (10)	0.0474 (11)	0.0429 (9)	0.0052 (9)	−0.0033 (8)	0.0027 (8)
C2	0.0427 (10)	0.0555 (12)	0.0628 (12)	0.0028 (9)	−0.0029 (9)	0.0008 (10)
C3	0.0404 (10)	0.0636 (13)	0.0547 (11)	−0.0071 (9)	0.0064 (8)	−0.0044 (9)
C4	0.0434 (10)	0.0597 (12)	0.0447 (10)	−0.0055 (9)	0.0030 (8)	0.0042 (9)
C5	0.0479 (10)	0.0453 (10)	0.0434 (10)	0.0053 (8)	0.0035 (8)	0.0041 (8)
C6	0.0441 (10)	0.0515 (11)	0.0447 (10)	0.0068 (8)	0.0050 (7)	0.0113 (8)
C7	0.0439 (10)	0.0746 (14)	0.0537 (11)	0.0018 (10)	0.0044 (8)	0.0000 (10)
C8	0.0549 (13)	0.0920 (19)	0.0722 (15)	−0.0139 (13)	−0.0054 (11)	0.0022 (13)
C9	0.0409 (12)	0.105 (2)	0.100 (2)	−0.0045 (13)	−0.0010 (12)	0.0259 (18)
C10	0.0473 (13)	0.094 (2)	0.107 (2)	0.0206 (13)	0.0232 (13)	0.0154 (17)
C11	0.0547 (12)	0.0673 (14)	0.0727 (14)	0.0151 (11)	0.0142 (10)	0.0038 (12)
C12	0.0482 (10)	0.0447 (10)	0.0454 (10)	0.0058 (8)	−0.0013 (8)	0.0049 (8)
C13	0.0555 (12)	0.0594 (13)	0.0593 (12)	0.0022 (10)	0.0044 (9)	−0.0082 (10)
C14	0.0709 (15)	0.0628 (15)	0.0828 (16)	0.0013 (13)	−0.0041 (13)	−0.0187 (13)
C15	0.0658 (15)	0.0561 (14)	0.108 (2)	−0.0080 (12)	−0.0114 (14)	−0.0023 (14)
C16	0.0580 (13)	0.0686 (16)	0.0944 (18)	−0.0040 (12)	0.0121 (12)	0.0157 (14)
C17	0.0595 (12)	0.0563 (13)	0.0621 (13)	0.0044 (11)	0.0102 (10)	0.0043 (10)
C18	0.0551 (11)	0.0497 (12)	0.0517 (11)	−0.0046 (10)	0.0037 (9)	−0.0045 (9)
C19	0.0647 (13)	0.0631 (13)	0.0458 (11)	−0.0097 (11)	−0.0023 (9)	−0.0083 (9)
C20	0.0583 (13)	0.100 (2)	0.0676 (15)	0.0072 (13)	0.0038 (11)	−0.0019 (14)
C21	0.0676 (16)	0.146 (3)	0.0796 (18)	−0.0136 (18)	0.0192 (14)	0.0062 (19)
C22	0.091 (2)	0.140 (3)	0.0686 (17)	−0.0126 (19)	0.0291 (15)	0.0074 (18)
C23	0.102 (2)	0.110 (2)	0.0614 (15)	0.0077 (19)	0.0183 (14)	0.0161 (15)
C24	0.0640 (14)	0.0943 (18)	0.0491 (12)	0.0090 (13)	0.0041 (10)	−0.0018 (12)
C25	0.0497 (11)	0.0903 (17)	0.0512 (12)	−0.0120 (12)	0.0019 (9)	0.0152 (11)
C26	0.105 (2)	0.141 (3)	0.0776 (18)	−0.011 (2)	−0.0199 (16)	0.061 (2)
N1	0.0521 (9)	0.0445 (9)	0.0413 (8)	0.0040 (7)	0.0006 (6)	0.0013 (7)
N2	0.0528 (10)	0.0681 (11)	0.0420 (8)	−0.0018 (9)	0.0040 (7)	−0.0044 (8)
O1	0.0955 (12)	0.0527 (9)	0.0690 (10)	0.0110 (9)	−0.0002 (9)	−0.0110 (8)
O2	0.0484 (8)	0.1009 (13)	0.0718 (10)	0.0021 (8)	0.0196 (7)	−0.0005 (9)
O3	0.0797 (12)	0.1485 (19)	0.0562 (10)	−0.0009 (12)	0.0214 (9)	0.0255 (11)
O4	0.0676 (10)	0.1049 (14)	0.0628 (10)	0.0032 (10)	0.0005 (8)	0.0414 (9)

### Geometric parameters (Å, °)

**Table:**

C27—C26	1.418 (4)	C14—H14	0.93
C27—H27A	0.96	C15—C16	1.376 (4)
C27—H27B	0.96	C15—H15	0.93
C27—H27C	0.96	C16—C17	1.379 (3)
C1—N1	1.473 (2)	C16—H16	0.93
C1—C2	1.524 (3)	C17—H17	0.93
C1—C12	1.528 (3)	C18—O1	1.221 (3)
C1—H1	0.98	C18—N1	1.361 (3)
C2—C3	1.481 (3)	C18—C19	1.528 (3)
C2—H2A	0.97	C19—N2	1.452 (3)
C2—H2B	0.97	C19—H19A	0.97
C3—O2	1.344 (2)	C19—H19B	0.97
C3—C4	1.352 (3)	C20—N2	1.463 (3)
C4—C25	1.446 (3)	C20—C21	1.514 (4)
C4—C5	1.516 (3)	C20—H20A	0.97
C5—N1	1.475 (2)	C20—H20B	0.97
C5—C6	1.530 (3)	C21—C22	1.509 (4)
C5—H5	0.98	C21—H21A	0.97
C6—C7	1.378 (3)	C21—H21B	0.97
C6—C11	1.386 (3)	C22—C23	1.508 (4)
C7—C8	1.392 (3)	C22—H22A	0.97
C7—H7	0.93	C22—H22B	0.97
C8—C9	1.367 (4)	C23—C24	1.510 (4)
C8—H8	0.93	C23—H23A	0.97
C9—C10	1.371 (4)	C23—H23B	0.97
C9—H9	0.93	C24—N2	1.465 (3)
C10—C11	1.384 (4)	C24—H24A	0.97
C10—H10	0.93	C24—H24B	0.97
C11—H11	0.93	C25—O3	1.220 (3)
C12—C13	1.381 (3)	C25—O4	1.335 (3)
C12—C17	1.388 (3)	C26—O4	1.448 (3)
C13—C14	1.385 (3)	C26—H26A	0.97
C13—H13	0.93	C26—H26B	0.97
C14—C15	1.369 (4)	O2—H2	0.82
			
C26—C27—H27A	109.5	C15—C16—H16	120.0
C26—C27—H27B	109.5	C17—C16—H16	120.0
H27A—C27—H27B	109.5	C16—C17—C12	121.2 (2)
C26—C27—H27C	109.5	C16—C17—H17	119.4
H27A—C27—H27C	109.5	C12—C17—H17	119.4
H27B—C27—H27C	109.5	O1—C18—N1	121.68 (19)
N1—C1—C2	108.30 (16)	O1—C18—C19	118.64 (19)
N1—C1—C12	112.21 (15)	N1—C18—C19	119.68 (18)
C2—C1—C12	114.98 (17)	N2—C19—C18	114.65 (17)
N1—C1—H1	107.0	N2—C19—H19A	108.6
C2—C1—H1	107.0	C18—C19—H19A	108.6
C12—C1—H1	107.0	N2—C19—H19B	108.6
C3—C2—C1	112.11 (16)	C18—C19—H19B	108.6
C3—C2—H2A	109.2	H19A—C19—H19B	107.6
C1—C2—H2A	109.2	N2—C20—C21	111.6 (2)
C3—C2—H2B	109.2	N2—C20—H20A	109.3
C1—C2—H2B	109.2	C21—C20—H20A	109.3
H2A—C2—H2B	107.9	N2—C20—H20B	109.3
O2—C3—C4	123.3 (2)	C21—C20—H20B	109.3
O2—C3—C2	113.52 (18)	H20A—C20—H20B	108.0
C4—C3—C2	123.13 (18)	C22—C21—C20	110.9 (3)
C3—C4—C25	119.49 (19)	C22—C21—H21A	109.5
C3—C4—C5	122.28 (17)	C20—C21—H21A	109.5
C25—C4—C5	117.99 (18)	C22—C21—H21B	109.5
N1—C5—C4	110.69 (15)	C20—C21—H21B	109.5
N1—C5—C6	112.96 (15)	H21A—C21—H21B	108.0
C4—C5—C6	114.55 (16)	C23—C22—C21	109.4 (2)
N1—C5—H5	106.0	C23—C22—H22A	109.8
C4—C5—H5	106.0	C21—C22—H22A	109.8
C6—C5—H5	106.0	C23—C22—H22B	109.8
C7—C6—C11	118.2 (2)	C21—C22—H22B	109.8
C7—C6—C5	122.69 (17)	H22A—C22—H22B	108.2
C11—C6—C5	119.04 (19)	C22—C23—C24	111.3 (3)
C6—C7—C8	121.1 (2)	C22—C23—H23A	109.4
C6—C7—H7	119.5	C24—C23—H23A	109.4
C8—C7—H7	119.5	C22—C23—H23B	109.4
C9—C8—C7	119.8 (3)	C24—C23—H23B	109.4
C9—C8—H8	120.1	H23A—C23—H23B	108.0
C7—C8—H8	120.1	N2—C24—C23	111.7 (2)
C8—C9—C10	119.9 (2)	N2—C24—H24A	109.3
C8—C9—H9	120.1	C23—C24—H24A	109.3
C10—C9—H9	120.1	N2—C24—H24B	109.3
C9—C10—C11	120.4 (2)	C23—C24—H24B	109.3
C9—C10—H10	119.8	H24A—C24—H24B	107.9
C11—C10—H10	119.8	O3—C25—O4	122.2 (2)
C10—C11—C6	120.6 (2)	O3—C25—C4	125.0 (2)
C10—C11—H11	119.7	O4—C25—C4	112.78 (19)
C6—C11—H11	119.7	C27—C26—O4	108.6 (3)
C13—C12—C17	117.8 (2)	C27—C26—H26A	110.0
C13—C12—C1	123.09 (18)	O4—C26—H26A	110.0
C17—C12—C1	118.96 (18)	C27—C26—H26B	110.0
C12—C13—C14	121.2 (2)	O4—C26—H26B	110.0
C12—C13—H13	119.4	H26A—C26—H26B	108.4
C14—C13—H13	119.4	C18—N1—C1	123.80 (16)
C15—C14—C13	120.1 (2)	C18—N1—C5	117.01 (16)
C15—C14—H14	120.0	C1—N1—C5	117.08 (14)
C13—C14—H14	120.0	C19—N2—C20	111.43 (19)
C14—C15—C16	119.8 (2)	C19—N2—C24	108.92 (17)
C14—C15—H15	120.1	C20—N2—C24	109.31 (17)
C16—C15—H15	120.1	C3—O2—H2	109.5
C15—C16—C17	120.0 (2)	C25—O4—C26	117.9 (2)
			
N1—C1—C2—C3	47.4 (2)	C13—C12—C17—C16	−0.6 (3)
C12—C1—C2—C3	−79.0 (2)	C1—C12—C17—C16	175.4 (2)
C1—C2—C3—O2	161.99 (18)	O1—C18—C19—N2	−112.8 (2)
C1—C2—C3—C4	−20.6 (3)	N1—C18—C19—N2	66.8 (3)
O2—C3—C4—C25	3.4 (3)	N2—C20—C21—C22	−57.8 (3)
C2—C3—C4—C25	−173.7 (2)	C20—C21—C22—C23	53.9 (4)
O2—C3—C4—C5	177.68 (19)	C21—C22—C23—C24	−53.7 (4)
C2—C3—C4—C5	0.6 (3)	C22—C23—C24—N2	57.3 (3)
C3—C4—C5—N1	−8.9 (3)	C3—C4—C25—O3	−8.8 (4)
C25—C4—C5—N1	165.46 (18)	C5—C4—C25—O3	176.7 (2)
C3—C4—C5—C6	120.3 (2)	C3—C4—C25—O4	169.4 (2)
C25—C4—C5—C6	−65.4 (2)	C5—C4—C25—O4	−5.0 (3)
N1—C5—C6—C7	106.2 (2)	O1—C18—N1—C1	−168.4 (2)
C4—C5—C6—C7	−21.8 (3)	C19—C18—N1—C1	12.1 (3)
N1—C5—C6—C11	−76.2 (2)	O1—C18—N1—C5	−5.5 (3)
C4—C5—C6—C11	155.77 (19)	C19—C18—N1—C5	175.05 (17)
C11—C6—C7—C8	−0.7 (3)	C2—C1—N1—C18	102.4 (2)
C5—C6—C7—C8	176.9 (2)	C12—C1—N1—C18	−129.59 (19)
C6—C7—C8—C9	0.4 (4)	C2—C1—N1—C5	−60.5 (2)
C7—C8—C9—C10	0.2 (4)	C12—C1—N1—C5	67.5 (2)
C8—C9—C10—C11	−0.5 (4)	C4—C5—N1—C18	−123.85 (19)
C9—C10—C11—C6	0.2 (4)	C6—C5—N1—C18	106.2 (2)
C7—C6—C11—C10	0.4 (3)	C4—C5—N1—C1	40.3 (2)
C5—C6—C11—C10	−177.3 (2)	C6—C5—N1—C1	−89.7 (2)
N1—C1—C12—C13	−125.6 (2)	C18—C19—N2—C20	58.5 (2)
C2—C1—C12—C13	−1.2 (3)	C18—C19—N2—C24	179.17 (19)
N1—C1—C12—C17	58.6 (2)	C21—C20—N2—C19	179.7 (2)
C2—C1—C12—C17	−177.07 (17)	C21—C20—N2—C24	59.2 (3)
C17—C12—C13—C14	0.2 (3)	C23—C24—N2—C19	179.1 (2)
C1—C12—C13—C14	−175.7 (2)	C23—C24—N2—C20	−58.9 (3)
C12—C13—C14—C15	0.6 (4)	O3—C25—O4—C26	−6.9 (4)
C13—C14—C15—C16	−0.9 (4)	C4—C25—O4—C26	174.8 (2)
C14—C15—C16—C17	0.5 (4)	C27—C26—O4—C25	−116.4 (5)
C15—C16—C17—C12	0.2 (4)		

### Hydrogen-bond geometry (Å, °)

**Table:**

*D*—H···*A*	*D*—H	H···*A*	*D*···*A*	*D*—H···*A*
O2—H2···O3	0.82	1.88	2.598 (3)	145
C2—H2B···O1^i^	0.97	2.43	3.286 (3)	148
C10—H10···O1^ii^	0.93	2.50	3.427 (3)	177

Symmetry codes: (i) −*x*, −*y*, −*z*; (ii) −*x*+1, −*y*, −*z*.

## References

[bb1] Aridoss, G., Amirthaganesan, S., Ashok Kumar, N., Kim, J. T., Lim, K. T., Kabilan, S. & Jeong, Y. T. (2008). *Bioorg. Med. Chem. Lett.* **18**, 6542–6548. 10.1016/j.bmcl.2008.10.045 18952418

[bb2] Aridoss, G., Amirthaganesan, S. & Jeong, Y. T. (2010). *Bioorg. Med. Chem. Lett.* **20**, 2242–2249. 10.1016/j.bmcl.2010.02.015 20207140

[bb3] Aridoss, G., Gayathri, D., Park, K. S., Kim, J. T. & Jeong, Y. T. (2009*a*). *Acta Cryst.* E**65**, o3180–o3181. 10.1107/S1600536809049186 PMC2971869 21578893

[bb4] Aridoss, G., Gayathri, D., Ramachandran, R., Lim, K. T. & Jeong, Y. T. (2009*b*). *Acta Cryst.* E**65**, o3232–o3233. 10.1107/S1600536809050259 PMC2971963 21578938

[bb5] Bruker (2008). *APEX2*, *SAINT* and *SADABS* Bruker AXS Inc., Madison, Wisconsin, USA.

[bb6] Cremer, D. & Pople, J. A. (1975). *J. Am. Chem. Soc.* **97**, 1354–1358.

[bb7] Farrugia, L. J. (1997). *J. Appl. Cryst.* **30**, 565.

[bb8] Nardelli, M. (1983). *Acta Cryst.* C**39**, 1141–1142.

[bb9] Parkin, A., Oswald, I. D. H. & Parsons, S. (2004). *Acta Cryst.* B**60**, 219–227. 10.1107/S0108768104003672 15017096

[bb10] Sheldrick, G. M. (2008). *Acta Cryst.* A**64**, 112–122. 10.1107/S0108767307043930 18156677

[bb11] Spek, A. L. (2009). *Acta Cryst.* D**65**, 148–155. 10.1107/S090744490804362X PMC2631630 19171970

[bb12] Subha Nandhini, M., Vijayakumar, V., Mostad, A., Sundaravadivelu, M. & Natarajan, S. (2003). *Acta Cryst.* E**59**, o1672–o1674.

